# Full-Length Sequence Analysis of Chloroquine Resistance Transporter Gene in *Plasmodium falciparum* Isolates from Sabah, Malaysia

**DOI:** 10.1155/2014/935846

**Published:** 2014-10-28

**Authors:** Lii Lian Tan, Tiek Ying Lau, William Timothy, Dhanaraj Prabakaran

**Affiliations:** ^1^Biotechnology Research Institute, Universiti Malaysia Sabah, Jalan UMS, 88400 Kota Kinabalu, Sabah, Malaysia; ^2^Infectious Diseases Unit, Queen Elizabeth Hospital, 88560 Kota Kinabalu, Sabah, Malaysia; ^3^Kudat District Hospital, 89057 Kudat, Sabah, Malaysia

## Abstract

Chloroquine resistance (CQR) in falciparum malaria was identified to be associated with several mutations in the chloroquine resistance transporter gene (*pfcrt*) that encodes the transmembrane transporter in digestive vacuole membrane of the parasite. This study aimed to investigate the point mutations across the full-length *pfcrt* in *Plasmodium falciparum* isolates in Sabah, Malaysia. A total of 31 *P. falciparum* positive samples collected from Keningau, Kota Kinabalu, and Kudat, Sabah, were analyzed. *pfcrt* was PCR amplified and cloned prior to sequence analysis. This study showed that all the previously described 10 point mutations associated with CQR at codons 72, 74, 75, 76, 97, 220, 271, 326, 356, and 371 were found with different prevalence. Besides, two novel point mutations, I166V and H273N, were identified with 22.5% and 19.3%, respectively. Three haplotypes, namely, CVMNK (29%), CVIET (3.2%), and SVMNT (67.7%), were identified. High prevalence of SVMNT among *P. falciparum* isolates from Sabah showed that these isolates are closer to the *P. falciparum* isolates from Papua New Guinea rather than to the more proximal Southeast Asian CVIET haplotype. Full-length analysis of *pfcrt* showed that chloroquine resistant *P. falciparum* in Sabah is still prevalent despite the withdrawal of chloroquine usage since 1979.

## 1. Introduction

Malaria is a disease caused by* Plasmodium* parasites that is transmitted by the female* Anopheles* mosquitoes. According to Asia Pacific Malaria Elimination Network (APMEN) Country Briefing-Malaysia 2012 [1],* P. falciparum*,* P. vivax*, and* P. knowlesi* were the most common* Plasmodium* species that contribute to the human malaria cases in Malaysia. The annual report of Malaysia Ministry of Health 2011 [2] stated that malaria remains a public health problem in Sabah due to the deep forest areas that house the vectors with 2032 cases reported in Sabah from a total of 5306 confirmed malaria cases reported throughout Malaysia in the year 2011.

The development of* Plasmodium* parasite toward the antimalarial drug has created the challenges for control and elimination of malaria. Chloroquine (CQ) was introduced widely throughout the world due to its effectiveness, low cost, and relative safety as compared to other antimalarial drugs [3]. In Malaysia, CQ was treated as the first-line treatment since early 1960s [4]. However, the first case of chloroquine resistant (CQR)* P. falciparum* was reported in 1963 [5] and subsequently more CQR cases were reported from Sabah, Sarawak, and Peninsular Malaysia [4, 6, 7]. Therefore, sulphadoxine-pyrimethamine (SDX/PYR) was later adopted as the first-line treatment for uncomplicated falciparum malaria in Peninsular Malaysia and Sabah in 1979 [8]. And, to date, artemisinin-combinational therapy (ACTs) is used as the first-line treatment for uncomplicated falciparum malaria.

The resistance of falciparum malaria toward CQ was identified to be associated with the mutations in the chloroquine resistance transporter gene (*pfcrt*) [9].* pfcrt* gene is a 3.1 kbp gene which comprises 13 exons which encode for 424-amino acid, 48.6 kDa protein that located at the digestive vacuole membrane of the parasite. The substitution of lysine (K) by threonine (T) at amino acid position 76 (K76T) has been identified as the primary marker conferring to CQR* in vitro* and* in vivo* [9, 10]. This molecular marker has been used as a useful tool for the surveillance of CQR in the field isolates.

Besides K76T, nine other point mutations at amino acid positions 72, 74, 75, 97, 220, 271, 326, 356, and 371 have been identified in* crt* gene of CQR* P. falciparum* in various combinations depending on the geographical origin of* P. falciparum* [9]. These additional mutations form the pattern that is specific to certain region in the world. CQR* P. falciparum* in Southeast Asia and Africa carry the point mutations at amino acid positions 74, 75, 76, 220, 271, 326, and 371, while CQR* P. falciparum* isolates from South America carry mutations at amino acid positions 76 and 220 with either combination of 72, 326, and 356 or combination of 75, 97, and 371 [9]. However, there is no association between numbers of mutations toward the level of CQR because it was suggested that other mechanisms on different loci are likely to modulate the level of CQR [11]. Besides that, polymorphisms at amino acid positions 72–76 have been associated with geographical origin of the parasites [9, 11]. Based on codons 72–76, haplotype CVIET represents the resistant isolates from Asia and Africa, and haplotype SVMNT is present in CQR isolates from Papua New Guinea and South America.

In Malaysia, 100% prevalence of K76T mutation was reported in Lundu, Sarawak, in the year 2003 [6], and 80.6% prevalence of K76T mutation was reported in Tawau, Sabah, in 2011 [3]. A study in Pahang, Peninsular Malaysia, in 2012 showed 52% prevalence of K76T, 7% for Q271E, 12% for N326S, 24% for I356T, and 77% for R371I [12]. Full-length analysis of* pfcrt* was conducted in this study to provide a thorough overview on the pattern of mutations of* pfcrt* and the prevalence of* P. falciparum* towards CQR in Sabah, Malaysia.

## 2. Methods

### 2.1. Study Sites

**Figure 1:**
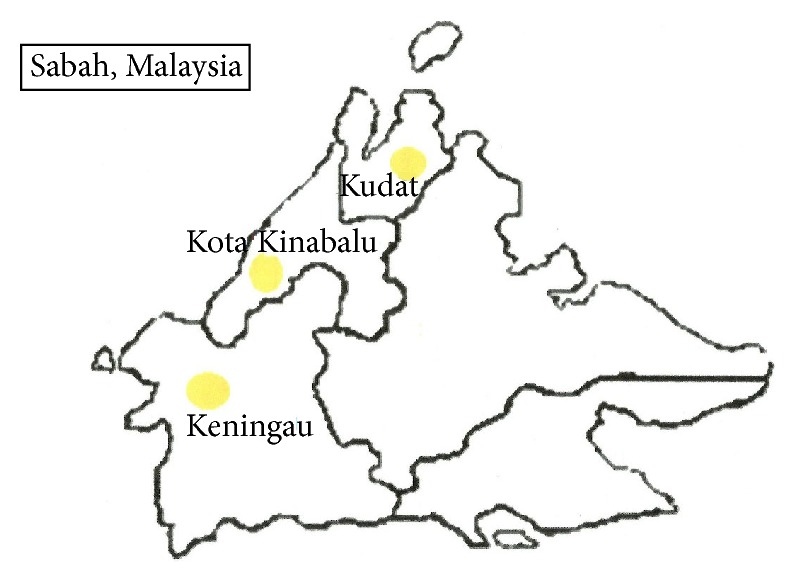
Map of study sites.

This study was carried out in the state of Sabah which is the second largest state in Malaysia that covers an area of 76,114.92 km^2^. Sabah is divided into five divisions, namely, West Coast, Kudat, Interior, Sandakan, and Tawau. Each of these divisions is subdivided into a total of 23 districts. Sabah is predominantly hilly and Sabah's forests cover an area of approximately 63% of its total landmass. In this study, the samples were collected from Kota Kinabalu, Kudat, and Keningau, Sabah (Figure 1).

### 2.2. Samples Collection

Ethical clearance was obtained from the Ministry of Health Malaysia to conduct this study. Patient's verbal and written consent were obtained prior to blood sample collection. Samples were collected in the year 2012 by the medical laboratory staffs from the respective hospitals. Patients attending Keningau District Hospital, Kudat District Hospital, and Queen Elizabeth Hospital who were suspected to be infected with malaria and had requested for blood film were recruited in this study. The blood samples were collected in the form of blood spots spotted on the 3 MM chromatography paper (Whatman 3 MM) with the volume of approximately 25 *μ*L each.

### 2.3. Genomic DNA Extraction

QIAamp DNA Mini Kit (QIAGEN, UK) was used to extract the genomic DNA from the dried blood spots according to the manufacturer's protocol. The extracted DNA was stored at −20°C prior to use.

### 2.4. Species-Specific Identification of Malaria Parasite by Nested PCR

*Plasmodium* species was identified by performing nested PCR using* Plasmodium* genus-specific and species-specific PCR based on small subunit ribosomal RNA (ssrRNA) gene [13].

### 2.5. PCR Amplification of* pfcrt*

**Figure 2:**
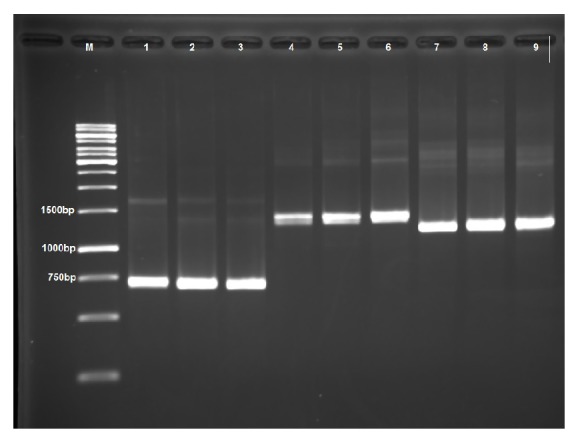
Agarose gel showing PCR product of* pfcrt* amplified in 3 fragments. Lane M: 1 kb DNA ladder; lanes 1 to 3: first fragment of* pfcrt* gene ~694 bp; lanes 4 to 6: second fragment of* pfcrt* gene ~1370 bp; and lanes 7 to 9: third fragment of* pfcrt* ~1235 bp.

PCR amplification of full-length* pfcrt* was performed in 31 single* P. falciparum* infection samples. Three independent nested PCRs were conducted to amplify full-length* pfcrt* gene (3.1 kb) in 3 fragments according to the protocol as previously described by Chaijaroenkul et al. [14] with some modifications. Primers used in the PCR were shown in Table 1. The PCR products were gel extracted using QIAquick Gel Extraction Kit (QIAGEN, UK) according to the manufacturer protocols prior to cloning (Figure 2).

### 2.6. Cloning of Fragment of* pfcrt*

*pfcrt* gene was cloned using CloneJET PCR Cloning Kit (Thermo Scientific, USA) according to the manufacturer's sticky-end cloning protocol with some modifications and was transformed into* Top10 E. coli*. Plasmids were purified by using QIAprep Spin Miniprep Kit (QIAGEN, UK) and digested with restriction enzymes* Xba*1 and* Xho*1 (New England Biolabs, UK) for confirmation of the correct insert prior to DNA sequencing.

### 2.7. Sequence Analysis

DNA sequencing was outsourced to Bioneer Corporation (Korea). The DNA sequencing service was performed on the ABI 3730XL DNA Analyzer using pJET1.2 forward and reverse sequencing primer. DNASTAR (Lasergene) and Mega 5.0 computer software was utilized for DNA sequence analysis.

## 3. Results

Full-length* pfcrt* gene for a total of 31* P. falciparum* isolates from Sabah which consisted of 1 isolate from Keningau (KG), 15 isolates from Kota Kinabalu (KK), and 15 isolates from Kudat (KT) was amplified in 3 fragments (see Figure 2) and analyzed. Based on the sequence analysis as referred to* pfcrt* of wild type 3D7 (GenBank: NC_004328.2), a total of 14 nonsynonymous amino acid substitutions at codons 72, 74, 75, 76, 144, 160, 166, 220, 271, 273, 326, 333, 356, and 371 were identified (see Table 2).

All the 10 point mutations with the exception of codon 97 that were previously reported to be associated with CQR were identified in* P. falciparum* isolates from this study [9]. Among the 9 point mutations, C72S, K76T, A220S, N326D, and I356L/T occur at the higher prevalence with 67.7%, 70.9%, 51.6%, 58%, and 51.6%, respectively, whereas M74I, N75E, Q271E, and R371I were observed only in one isolate with 3.2% of prevalence (see Table 2). Only one sample (KG007) showed 7 out of the 10 point mutations which is equivalent to the CQR* P. falciparum* isolates from Southeast Asia region, forming the pattern C*IET*H*SE*N*TI* from amino acid position 72 to 371. Besides, 15 samples with 5 point mutations giving pattern of  *S*MN*T*H*S*Q*DL*R were similar to the CQR falciparum from South America and Papua New Guinea. The remaining 15 samples showed less than 3 point mutations (C72S, K76T, or N326D) which are lower than the reported minimum four point mutations in* pfcrt* to confer CQR parasite [15].

A144T and L160Y that were previously reported to be found only in the Philippines [16] were also detected in 7 out of 31 study isolates (22.5%). In addition, point mutation at codon 333 that was previously reported in Cambodia (T333S) by Durrand et al. [17] was also detected in 48.3% (15 out of 31) of* P. falciparum* isolates in this study but with different amino acid substitution (T333A). In addition, 2 novel point mutations, I166V and H273N, were identified in this study with the prevalence 22.5% for I166V and 19.3% for H273N.

Sequence polymorphism of* pfcrt* in positions 72–76 showed that three types of* pfcrt* haplotypes, namely, S_agt_VMNT, CVIET, and CVMNK, were observed in this study. CQR type S_agt_VMNT was predominant (21 out of 31, 67.7%) while only 1 sample from Keningau with CVIET haplotype (3.2%) and the rest of the* P. falciparum* isolates were of CQ susceptible type CVMNK (9 out of 31, 29%) (see Table 3).

## 4. Discussion

In Sabah, Malaysia, CQR* P. falciparum* were reported in 1971-1972. Due to the high prevalence of resistance, Fansidar (sulfadoxine/pyrimethamine) had replaced CQ for* P. falciparum* treatment in 1979 throughout the state of Sabah [18]. To date, only one study reported high prevalence (81%) of* pfcrt* K76T mutation in Tawau, Sabah, in 2011 [3]. K76T has been shown to be highly associated with CQR and acts as key marker of* P. falciparum* CQR. It had been reported that a lysine to threonine substitution at position 76 (K76T) was found in both* in vitro* and* in vivo* studies from* P. falciparum* in malaria endemic countries [9, 19]. 71% or 22 out of 31* P. falciparum* isolates from Sabah carrying K76T mutation reflected a high prevalence of CQR despite the discontinuation of CQ as first-line* P. falciparum* treatment in Sabah since 1979. Besides that, previous studies conducted in other states in Malaysia also reflected a high prevalence of CQR based on K76T marker with 100% and 52% in Lundu, Sarawak, in 2003 and Pahang in 2012, respectively [6, 12]. Besides, high prevalence of* pfcrt* K76T mutation is also in accordance with studies conducted in several neighbouring countries such as Indonesia, Thailand, and Philippines [20–22].

Based on 10 point mutations across amino acid positions 72 to 356 that were previously reported by Fidock et al. [9] to be associated with CQR, a combination of at least four mutational events in* pfcrt* including K76T must be present in order to associate the field isolates to CQR [15]. Our findings showed that 15 out of 31 isolates showed no less than 4 mutational events which might indicate that these isolates were associated to CQR (see Table 2). In addition, seven to nine point mutations were observed in CQR* P. falciparum* isolates from Southeast Asia and Africa forming the haplotype pattern of C*IET*H(*L*)*SEST*(I)*I* whereas four to five point mutations were observed in CQR* P. falciparum* from South America and Papua New Guinea forming haplotype pattern of  *S*MN*T*H*S*Q*DL*R [11, 15, 16]. Most of our* P. falciparum* isolates (14 out of 31) showed five of the described point mutations from South America and Papua New Guinea. Hence CQR* P. falciparum* from Sabah isolates are similar to CQR* P. falciparum* from South America and Papua New Guinea.

Besides that, only 1 isolate (KG007) from this study showed mutation at codons 74, 75, 271, and 371 (see Table 2). This isolate showed haplotype pattern of C*IET*H*SE*N*TI* with 7 out of 10 point mutations similar to that observed in CQR* P. falciparum* from Southeast Asia and Africa [15]. This finding suggested that this isolate might have originated from Southeast Asian countries such as Cambodia and Thailand and spread to the island of Borneo [23]. As for mutation at codon 271, similar result was observed in study by Atroosh et al. [12] in Pahang, Malaysia, whereby 93% of the study isolates were of wild type. Contrarily, Pahang isolates showed a high prevalence (77%) of R371I. Most of the* P. falciparum* isolates from Sabah (30 out of 31 isolates) show wild type R371. It is reported that the presence of mutations M74I, N75E, Q271E, and R371I has correlation with CQR; however the role of these mutations in conferring CQR is still unclear and it was suggested that these mutations may require maintaining the native* pfcrt* function [10, 17].

The dominance of the SVMNT over CVIET CQR associated haplotype (21 to 1) showed that the CQR* P. falciparum* in this region is closely related to CQR isolates originating from South America and Papua New Guinea rather than Southeast Asia. Only one isolate (KG007) in this study showed CVIET haplotype which was reported to be found in parasites in Africa, Southeast Asia, and South America [24]. There are two types of amino acid encoding serine (S) in amino acid position 72 in the SVMNT haplotype. Namely S_tct_VMNT from South America or S_agt_VMNT from Papua New Guinea [25]. Therefore, Sabah which is located nearer to Papua New Guinea is common to have the CQR isolates with S_agt_VMNT haplotypes. In addition, S_agt_VMNT haplotypes were also dominant in Lombok Indonesia and Philippines [26, 27]. Wootton et al. [15] proposed that CQR* P. falciparum* originated from at least four independent foci, namely, Asia, Papua New Guinea, Peru, and Colombia (South America). Therefore, it is anticipated that CQR isolates in Sabah might be originated from Papua New Guinea.

Interestingly, the currently described A144T and L160Y mutations in* pfcrt* that were only found in Philippines were also detected among the* P. falciparum* isolates from Sabah. According to Chen et al. [16], A144T and L160Y in Philippines were observed in combination with two or three mutated codons (K76T/326D or C72S/K76T/N326D) forming the CQR allelic type (K76T/A144T/L160Y/N326D). It had been suggested that these two novel mutations evolved independently in the Philippines and existed outside the 10 codons found in CQR isolates from Asia, Africa, Papua New Guinea, and South America [16]. In our study, 7 isolates showed these two point mutations. However, only one sample (KT066) formed the CQR allelic type similar to the* P. falciparum* isolates from the Philippines. The remaining 6 isolates carry A144T and L160Y mutations together with different combinations of mutated codons. Among the 6 samples, 2 samples showed these mutations together with K76T and C72S and the remaining 4 isolates having these mutations independently without combination with other known point mutations. Chen et al. [16] reported that* pfcrt* allelic types K76T/N326D/A144T/L160Y were resistant to CQ and desethyl CQ when tested* in vitro*. In addition, Chen et al. [16] also reported that these* pfcrt* allelic types have K76T mutation but not A220S mutation, which lead to the hypothesis that A144T and L160Y may be compensatory mutations that can confer CQR in the absence of A220S. However, A144T and L160Y mutations in conferring CQR are not clear [28]. Similar report was obtained from this study at which the* P. falciparum* isolates with these allelic types have the K76T mutation but not A220S mutation. Interestingly, mutations at A144T and L160Y were only observed in the isolates collected from Kudat, Sabah, but not in isolates from Kota Kinabalu and Keningau. So this may suggest that* P. falciparum* isolates carrying A144T and L160Y might spread from the Philippines to Kudat as the district located near to the Philippines. Besides that, Chen et al. [16] suggested that the A144T and L160Y mutations may occur in CQ-sensitive parasites that were later inherited to falciparum parasites that developed CQR. This is in accordance with the finding in this study because the A144T and L160Y mutations in this study do occur independently without any of the known point mutations that confer CQR.

Besides L160Y mutation that was reported from the Philippines, a different amino acid substitution forming L160P was found in one of the* P. falciparum* isolates in this study. Similarly, in the case of A144T that was reported from the Philippines, Durrand et al. [17] reported A144F in Cambodia and explained that this difference in amino acid change at a similar point mutation indicates that the* pfcrt* gene in CQR isolates evolves differently based on geographical areas. Durrand et al. [17] also reported that* pfcrt* sequences propagate in various epidemiological contexts which might be influenced by the genetic characteristics of the host, selection pressure due to the drug, and the intensity of transmission leading to the production of protein that is more likely to ensure the survival of the parasite under sustained and diverse drug selection pressures.

On the other hand, all the seven* P. falciparum* isolates carrying A144T and L160Y in this study have a novel mutation, I166V. Mutations A144T, L160Y, and I166V were observed to occur in combination either with or without K76T and N326D. Hence, further investigation is needed to determine the relation or role of these mutations in conferring CQR or is due to the mutations that occur in CQ-sensitive parasites that were then developed CQR and inherit this point mutation to their next generation. Besides, novel mutation I166V might arise independently in Sabah isolates giving rise to a haplotype specific to Sabah. On top of that, another novel mutation H273N was observed in 6 out of 31 study isolates. Four of these isolates carry H273N together with K76T. Interestingly, one isolate (KT070) carried H273N mutation alone across the whole* pfcrt* gene.

Durrand et al. [17] reported that mutation at position 333 (T333S) was only found in Cambodian isolates. Interestingly, mutation at this codon was also found in* P. falciparum* isolates from Kota Kinabalu, Sabah, but with T333A. The difference in amino acid substitution again indicates that* pfcrt* gene in CQR isolates evolves differently based on geographical areas. In our study, 14 of 15 Kota Kinabalu isolates carry T333A in combination with K76T, A220S, N326D, and I356L that were associated with CQR. Therefore, T333A mutation might play a role in CQR and may act as additional marker for CQR parasite identification together with the 10 reported codons.

## 5. Conclusions

This preliminary data showed that CQR* P. falciparum* is still prevalent in Sabah despite discontinuation of CQ as first-line treatment for* P. falciparum* since 1979 [18]. This can be supported by Sá et al. [29] which reported that CQR haplotype of SVMNT which is dominant in this study (67.7%) does not revert back to CQ sensitive although CQ is withdrawn. Although* in vivo* drug efficacy assessment is the gold standard for monitoring drug resistance for controlling malaria, the molecular marker and* in vitro* test can also provide useful information for drug resistance surveillance. Full-length amplification of* pfcrt* among* P. falciparum* isolates from Sabah will provide a more thorough analysis of the CQ drug resistance molecular marker in this geographical region.

## Figures and Tables

**Table 1:** Primers for *pfcrt* PCR amplification.

Primer	Primer sequence	Expected size (bp)	Reference
First fragment			
E1/2-F E1/2-R	5′-CGACATTCCGATATATTATATTTTAGAC-3′ 5′-TATATGTGTAATGTTTTATATTGG-3′	740	[14]
E1/2-NF E1/2-NR	5′-CCGTTAATAATAAATACACGCAG-3′ 5′-AATGTTTTATATTGGTAGGTGG-3′	694	
Second fragment			
E3/8-F E3/8-R	5′-CCACCTACCAATATAAAACATTAC-3′ 5′-GTTAAAATATATATAAATGTCTC-3′	1446	
E3/8-NF	5′-TATATATATATGTATGTATGTTG-3′	1370	
E3/8-NR	5′-AATGTCTCTTATAATTTTGAAATT-3′		
Third fragment			
E9/13-F	5′-CTTATAATAAAATTTCAAAATTATAAGAGAC-3′	1287
E9/13-R	5′-GAGATCTCTATACCTTCAACATTATTCC-3′	
E9/13-NF E9/13-NR	5′-GAGACATTTATATATATTTTAAC-3′ 5′-CCTTATAAAGTGTAATGCG-3′	1235	

**Table 2:** Analysis of point mutation in *pfcrt *among *P. falciparum *isolates from Sabah.

	Amino acid position													
	72	74	75	76	144	160	**166**	220	271	**273**	326	333	356	371
3D7 [GenBank NC_004328.2]	C	M	N	K	A	L	**I**	A	Q	**H**	N	T	I	R
KG007	*·*	I	E	T	*·*	*·*	***·***	S	E	***·***	*·*	*·*	T	I
KK004	*·*	*·*	*·*	*·*	*·*	*·*	***·***	S	*·*	***·***	D	A	L	*·*
KK005	S_agt_	*·*	*·*	T	*·*	*·*	***·***	S	*·*	***·***	D	A	L	*·*
KK007	S_agt_	*·*	*·*	T	*·*	*·*	***·***	S	*·*	***·***	D	A	L	*·*
KK008	S_agt_	*·*	*·*	T	*·*	*·*	***·***	S	*·*	***·***	D	A	L	*·*
KK012	S_agt_	*·*	*·*	T	*·*	*·*	***·***	S	*·*	***·***	D	A	L	*·*
KK020	S_agt_	*·*	*·*	T	*·*	*·*	***·***	S	*·*	***·***	D	A	L	*·*
KK022	S_agt_	*·*	*·*	T	*·*	*·*	***·***	S	*·*	***·***	D	A	L	*·*
KK023	S_agt_	*·*	*·*	T	*·*	*·*	***·***	S	*·*	***·***	D	A	L	*·*
KK025	S_agt_	*·*	*·*	T	*·*	*·*	***·***	S	*·*	***·***	D	A	L	*·*
KK026	S_agt_	*·*	*·*	T	*·*	*·*	***·***	S	*·*	***·***	D	A	L	*·*
KK027	S_agt_	*·*	*·*	T	*·*	*·*	***·***	S	*·*	***·***	D	A	L	*·*
KK031	S_agt_	*·*	*·*	T	*·*	*·*	***·***	S	*·*	***·***	D	A	L	*·*
KK032	S_agt_	*·*	*·*	T	*·*	*·*	***·***	S	*·*	***·***	D	A	L	*·*
KK037	S_agt_	*·*	*·*	T	*·*	*·*	***·***	S	*·*	***·***	D	A	L	*·*
KK039	S_agt_	*·*	*·*	T	*·*	*·*	***·***	S	*·*	***·***	D	A	L	*·*
KT038	S_agt_	*·*	*·*	T	*·*	*·*	***·***	*·*	*·*	***·***	*·*	*·*	*·*	*·*
KT052	*·*	*·*	*·*	*·*	T	Y	**V**	*·*	*·*	***·***	*·*	*·*	*·*	*·*
KT055	S_agt_	*·*	*·*	T	T	Y	**V**	*·*	*·*	**N**	*·*	*·*	*·*	*·*
KT066	S_agt_	*·*	*·*	T	T	Y	**V**	*·*	*·*	***·***	D	*·*	*·*	*·*
KT069	*·*	*·*	*·*	*·*	T	Y	**V**	*·*	*·*	***·***	*·*	*·*	*·*	*·*
KT070	*·*	*·*	*·*	*·*	*·*	*·*	***·***	*·*	*·*	**N**	*·*	*·*	*·*	*·*
KT072	S_agt_	*·*	*·*	T	T	Y	**V**	*·*	*·*	***·***	*·*	*·*	*·*	*·*
KT081	*·*	*·*	*·*	*·*	*·*	*·*	***·***	*·*	*·*	***·***	*·*	*·*	*·*	*·*
KT085	*·*	*·*	*·*	*·*	T	Y	**V**	*·*	*·*	***·***	*·*	*·*	*·*	*·*
KT088	S_agt_	*·*	*·*	T	*·*	*·*	***·***	*·*	*·*	**N**	*·*	*·*	*·*	*·*
KT092	*·*	*·*	*·*	*·*	*·*	*·*	***·***	*·*	*·*	***·***	*·*	*·*	*·*	*·*
KT094	S_agt_	*·*	*·*	T	*·*	P	***·***	*·*	*·*	**N**	*·*	*·*	*·*	*·*
KT096	S_agt_	*·*	*·*	T	*·*	*·*	***·***	*·*	*·*	**N**	D	*·*	*·*	*·*
KT097	*·*	*·*	*·*	*·*	T	Y	**V**	*·*	*·*	**N**	*·*	*·*	*·*	*·*
KT099	*·*	*·*	*·*	*·*	*·*	*·*	***·***	*·*	*·*	***·***	D	*·*	*·*	*·*

“*·*” indicates similar nucleotide as compared to the wild type 3D7; bold font indicates the novel point mutation.

**Table 3:** Prevalence of *pfcrt* haplotypes.

	Haplotype (72–76)	Number of samples (*n* = 31)	Percentage of prevalence (%)
CQ resistant	S_agt_MNT	21	67.74
	CVIET	1	3.23

CQ sensitive	CVMNK	9	29.03
